# Benzyl Isothiocyanate Attenuates Inflammasome Activation in *Pseudomonas aeruginosa* LPS-Stimulated THP-1 Cells and Exerts Regulation through the MAPKs/NF-κB Pathway

**DOI:** 10.3390/ijms23031228

**Published:** 2022-01-22

**Authors:** Won Sun Park, Jeonghan Lee, Giyoun Na, SaeGwang Park, Su-Kil Seo, Jung Sik Choi, Won-Kyo Jung, Il-Whan Choi

**Affiliations:** 1 Department of Physiology, Kangwon National University School of Medicine, Chuncheon 24341, Korea; parkws@kangwon.ac.kr; 2 Department of Anesthesiology and Pain Medicine, Busan Paik Hospital, Inje University, Busan 47392, Korea; ljh646@hanmail.net; 3 Department of Microbiology and Immunology, College of Medicine, Inje University, Busan 47392, Korea; gyna80@inje.ac.kr (G.N.); micpsg@inje.ac.kr (S.P.); sseo@inje.ac.kr (S.-K.S.); 4 Department of Internal Medicine, Busan Paik Hospital, Inje University, Busan 47392, Korea; cwj1225@naver.com; 5 Department of Biomedical Engineering, Center for Marine-Integrated Biomedical Technology (BK21 Plus), Pukyong National University, Busan 48513, Korea; wkjung@pknu.ac.kr

**Keywords:** inflammasomes, benzyl isothiocyanate, interleukin-1β, *Pseudomonas aeruginosa*, THP-1 cells, nuclear factor-κB

## Abstract

Inflammasomes are a group of intracellular multiprotein platforms that play important roles in immune systems. Benzyl isothiocyanate (BITC) is a constituent of cruciferous plants and has been confirmed to exhibit various biological activities. The modulatory effects of BITC on inflammasome-mediated interleukin (IL)-1β expression and its regulatory mechanisms in *Pseudomonas aeruginosa* (*P. aeruginosa*) LPS/ATP-stimulated THP-1 cells was investigated. Monocytic THP-1 cells were treated with phorbol myristate acetate (PMA) to induce differentiation into macrophages. Enzyme-linked immunosorbent assays (ELISA) were performed to measure the levels of IL-1β produced in *P. aeruginosa* LPS/ATP-exposed THP-1 cells. Western blotting was performed to examine the BITC modulatory mechanisms in inflammasome-mediated signaling pathways. BITC inhibited IL-1β production in *P. aeruginosa* LPS/ATP-induced THP-1 cells. BITC also inhibited activation of leucine-rich repeat protein-3 (NLRP3) and caspase-1 in *P. aeruginosa* LPS/ATP-induced THP-1 cells. Furthermore, we show that mitogen-activated protein kinase (MAPK) and nuclear factor-κB (NF-κB) activation in *P. aeruginosa* LPS was attenuated by BITC. These BITC-mediated modulatory effects on IL-1β production may have therapeutic potential for inflammasome-mediated disorders such as a nasal polyp.

## 1. Introduction

Interleukin-1β (IL-1β) is a critical regulator associated with acute and chronic inflammatory responses. IL-1β plays an important role in the pathogenesis of microbial infection and tissue injury, affecting nearly all tissues and organs in the host. Augmented levels of IL-1β have been linked to a broad spectrum of health and disease [1]. Like a double-edged sword, IL-1β is generally essential for normal host defense systems, but failure to control IL-1β destroys tissues and organs. IL-1β is synthesized as a precursor protein (pro-IL-1β) in the cytosol in response to diverse exogenous or endogenous stimuli [2]. Pro-IL-1β must be cleaved by activated caspase-1, which results in the maturation and secretion of IL-1β in LPS-stimulated cells. Caspase-1 is activated by an assembly of intracellular multiprotein complexes called inflammasomes [3]. Inflammasomes contain a nucleotide-binding domain and leucine-rich repeat protein-3 (NLRP3) sensor, an apoptosis-associated speck-like protein containing a caspase-recruitment domain (ASC) adaptor, and a caspase-1 enzyme (pro-caspase-1) [4,5,6]. It is well known that inflammasomes are activated in macrophages by the immune response to pathogen infections [5]. Generally, the common activation mechanism of inflammasome assembly is pro-caspase-1 associated with NLRP3 binding to the ASC. Then, pro-caspase-1 is activated to caspase-1 and expresses IL-1β as an alarm signal, and IL-1β triggers various mediators, resulting in activation of the immune system, for example, the inflammatory response [7]. Therefore, the activation of inflammasome components represents an important intracellular signaling pathway in inflammatory responses.

**Figure 1:**
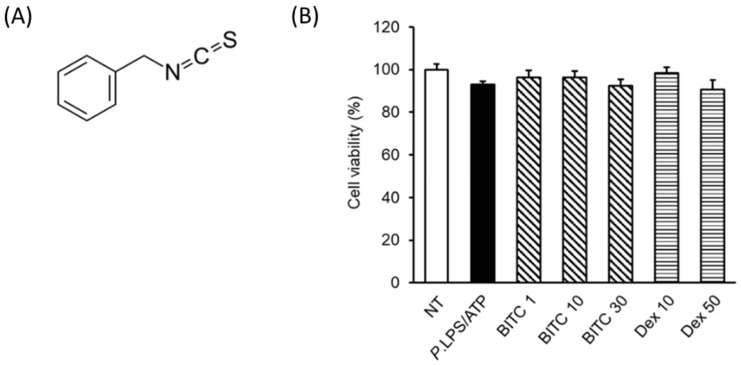
The chemical structure of BITC and the effect of BITC on THP-1 cell viability. (**A**) Chemical structure of BITC. (**B**) THP-1 cells were treated with various BITC (1, 10, and 30 μM) and Dex (10 and 50 μM) concentrations for 24 h. Cell viability was assessed using the CCK-8 assay, and the results are expressed as the percentage of surviving cells relative to the untreated *P. aeruginosa* LPS cells. Each value indicates the mean ± SD and is representative of results obtained from three independent experiments. NT, non-treated; Dex, dexamethasone; *P*. LPS, *P. aeruginosa* LPS.

Isothiocyanates (ITCs) are phytochemicals abundant in cruciferous vegetables, including broccoli, radishes, cabbage, mustard greens, kale, cauliflower, bok choy, and others that are commonly eaten [8]. ITCs have been used as potent chemopreventive agents with pharmacological activities and possess health advantages and efficacy in the control of various human disorders, such as neurodegenerative diseases, cardiovascular diseases, cancer, and diabetes [9]. ITC groups, such as sulforaphane, moringin, phenethyl isothiocyanate (PITC), allyl isothiocyanate (AITC), phenyl ethyl isothiocyanate (PEITC), and benzyl isothiocyanate (BITC), are derived from the hydrolysis of glucosinolates [10]. Among isothiocyanate groups, BITC (C8H7NS, Figure 1A) exerts antioxidant, anticancer, anti-inflammatory, antimicrobial, and immunomodulatory effects [11,12,13,14,15]. Although some reports have mentioned the anti-inflammatory effect of BITC through inflammasome modulation [16,17], there is no information on the molecular mechanisms of the anti-inflammatory effects of BITCs on macrophages in the nasal polyp environment. A previous study showed that ITCs exert antimicrobial activity on *Pseudomonas aeruginosa* (*P. aeruginosa*) growth [18]. The present study focused on the BITC-mediated attenuation of *P. aeruginosa* LPS-stimulated IL-1β production by inhibiting the activation of inflammasome components through the reduction in specific signaling pathways in macrophages. Based on these results, we wanted to know whether BITC can be a therapeutic agent for a wide range of inflammatory disorders, especially nasal polyps.

## 2. Results

### 2.1. Effects of BITC on THP-1 Cell Viability

Initially, we evaluated the viability of THP-1 cells treated with BITC (Figure 1B). Dex (10 and 50 μM) was used as a positive control. Treatment of THP-1 cells with up to 30 μM BITC did not affect cell viability.

### 2.2. Effects of BITC on IL-1β Expression in P. aeruginosa LPS/ATP-Induced THP-1 Cells

**Figure 2:**
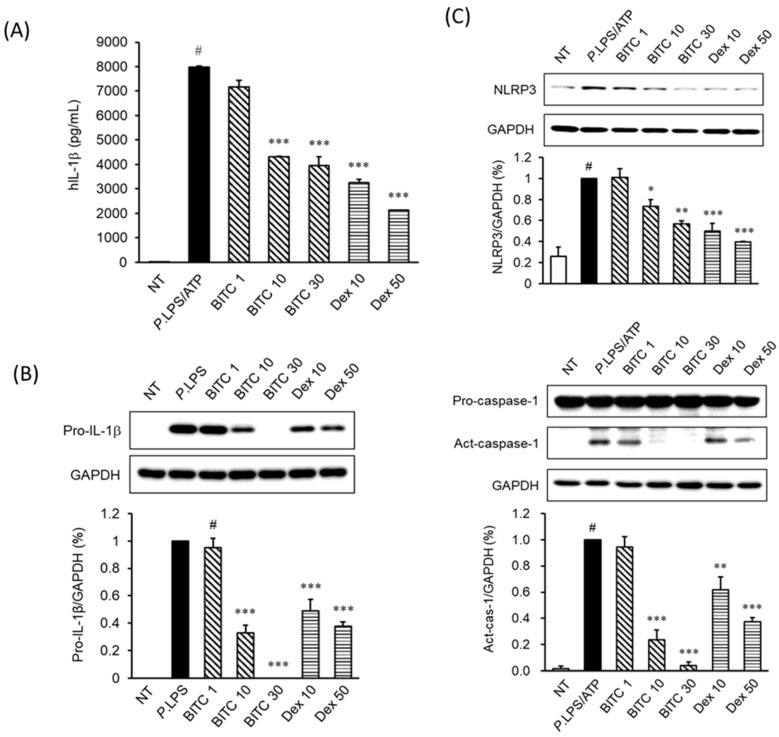
Effects of BITC on IL-1β production and inflammasome activation in *P. aeruginosa* LPS/ATP-stimulated primed THP-1 cells. The cells were seeded at 1 × 10^6^ cells/mL and incubated with various concentrations (1, 10, and 30 μM) of BITC for 1 h, followed by *P. aeruginosa* LPS stimulation (10 μg/mL). The cells were additionally stimulated with 5 mM ATP for 30 min. After stimulation with *P. aeruginosa* LPS, IL-1β production (**A**, LPS for 24 h) and pro-IL-1β expression (**B**, LPS for 3 h) were determined via ELISA and western blotting, respectively. (**C**) After treatment with LPS/ATP in the presence or absence of BITC for 1 h, NLRP3 and caspase-1 protein expression were determined using western blotting. Untreated cells were used as a control. Each value indicates the mean ± SD and is representative of results obtained from three independent experiments. # *p* < 0.05 vs. untreated group (NT); * *p* < 0.05, ** *p* < 0.01, *** *p* < 0.001 vs. *P. aeruginosa* LPS group.

To evaluate the inhibitory effect of BITC on *P. aeruginosa* LPS/ATP-stimulated IL-1β expression in THP-1 cells, IL-1β expression levels in cell culture media were measured. As shown in Figure 2A, treatment with *P. aeruginosa* LPS/ATP markedly increased IL-1β release from THP-1 cells. However, pretreatment with BITC prevented this increase in the levels of IL-1β expression in *P. aeruginosa* LPS/ATP-stimulated THP-1 cells. A significantly reduced level of IL-1β was observed at both 10 and 30 µM BITC. To secrete IL-1β requires the expression of pro-IL-1β followed by its proteolytic processing by the active caspase-1. *P. aeruginosa* LPS strongly induced pro-IL-1β expression, but BITC treatment significantly decreased the amount of pro-IL-1β in THP-1 cells (Figure 2B).

### 2.3. Effects of BITC on NLRP3 and Caspase-1 Expression in P. aeruginosa LPS/ATP-Stimulated THP-1 Macrophages

THP-1 cells were stimulated with *P. aeruginosa* LPS/ATP in the presence or absence of BITC (1, 10 and 30 μM). As shown in Figure 2C, to investigate the effect of BITC on NLRP3 and active caspase-1 expression in LPS/ATP-induced THP-1 cells, we pretreated cells with BITC before stimulation with *P. aeruginosa* LPS/ATP. BITC treatment suppressed LPS/ATP-induced NLRP3 production and Act-caspase-1 (subunit p10) activation.

### 2.4. Effects of BITC on the Phosphorylation of the MAPK Signaling Pathways in P. aeruginosa LPS-Stimulated THP-1 Macrophages

**Figure 3:**
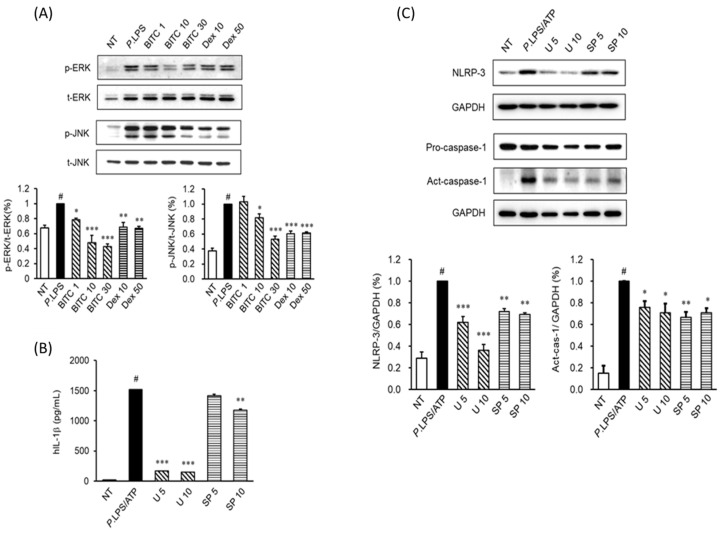
The effects of BITC on inflammasome activation in *P. aeruginosa* LPS-stimulated THP-1 cells. The cells were seeded at 1 × 10^6^ cells/mL and incubated with various BITC concentrations (1, 10, and 30 μM) for 1 h prior to *P. aeruginosa* LPS stimulation (10 μg/mL). (**A**) After treatment with *P. aeruginosa* LPS for 15 min and 30 min, the phosphorylated forms of ERK and JNK were determined using western blotting, respectively. (**B**) After stimulation with *P. aeruginosa* LPS/ATP for 24 h in the presence or absence of U 0126 and SP 600126 for 1 h, IL-1β production by THP-1 cells was determined via ELISA. (**C**) After treatment with LPS/ATP in the presence or absence of U 0126 (5 and 10 μM) and SP 600126 (5 and 10 μM) for 1 h, NLRP3 and caspase-1 protein expression were determined using western blotting. GAPDH was used as an internal control for the western blot analyses. Each value indicates the mean ± SD and is representative of results obtained from three independent experiments. # *p* < 0.05 vs. untreated group; * *p* < 0.05, ** *p* < 0.01 and *** *p* < 0.001 vs. *P. aeruginosa* LPS group. NT, untreated group; U, U 0126; SP, SP 600126.

To clarify the signaling pathways underlying the attenuation effects of BITC on IL-1β production and inflammasome component activation, we inspected the phosphorylation of mitogen-activated protein kinases (MAPKs). As a result of stimulation with *P. aeruginosa* LPS, phosphorylation of JNK and ERK was increased. The levels of JNK and ERK phosphorylation were highest at 15 and 30 min after incubation with *P. aeruginosa* LPS, respectively. However, pretreatment for 1 h with BITC (10 and 30 µM) attenuated the phosphorylation of JNK and ERK (Figure 3A). To demonstrate whether the ERK and JNK signaling pathways are involved in IL-1β production and inflammasome component activation, THP-1 cells were stimulated with *P. aeruginosa* LPS/ATP with or without ERK (U 0126)- and JNK (SP 600126)-specific inhibitors. The upregulated production of IL-1β (Figure 3B) and activation of inflammasome components (Figure 3C) were significantly attenuated by U 0126 and SP 600126. This result showed that BITC suppresses IL-1β production and inflammasome activation by inhibiting the ERK and JNK signaling pathways.

### 2.5. The Effects of BITC on NF-κB Activation in LPS-Stimulated THP-1 Macrophages

**Figure 4:**
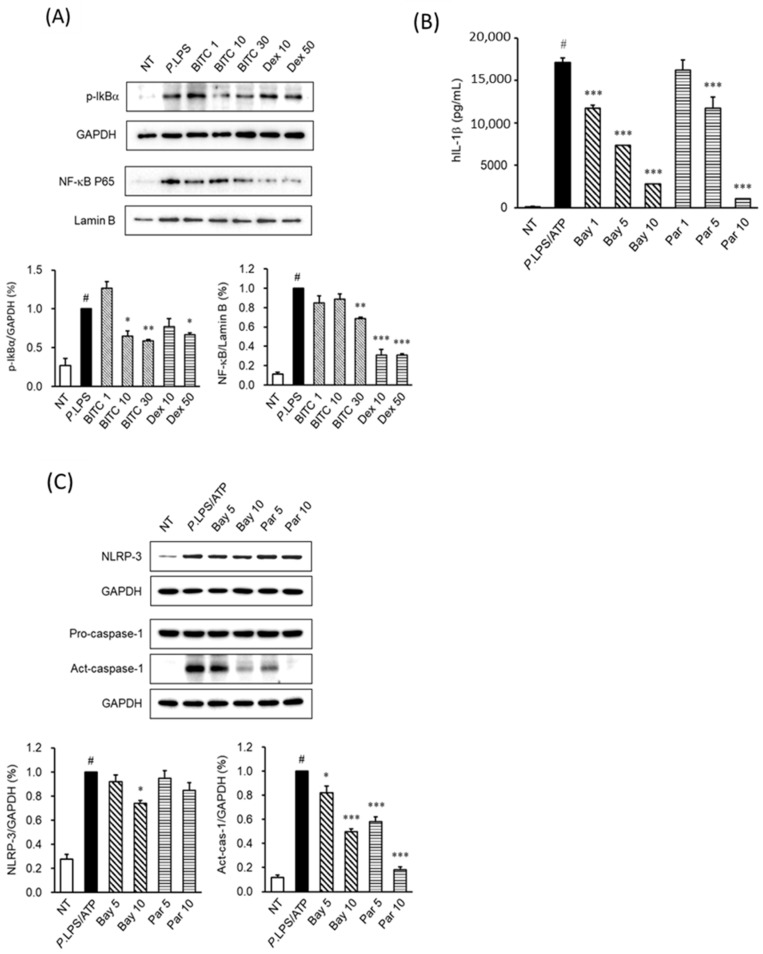
The effect of BITC on NF-κB activation in *P. aeruginosa* LPS-stimulated THP-1 cells. (**A**) The cells were pretreated with BITC (1, 10, and 30 μM) for 1 h and then stimulated with *P. aeruginosa* LPS for 1 h. Cytosol protein extracts were prepared as described in the materials and methods and evaluated for phosphorylation of IkBα using western blot analyses. Nuclear protein extracts were prepared and analyzed for NF-κB nuclear translocation using western blot analyses. (**B**) After stimulation with *P. aeruginosa* LPS/ATP for 24 h in the presence or absence of Bay 11-7082 (1, 5, and 10 μM) and parthenolide (1, 5, and 10 μM) for 1 h, IL-1β production by THP-1 cells was determined via ELISA. (**C**) After treatment with LPS/ATP in the presence or absence of Bay 11-7082 and parthenolide for 1 h, NLRP3 (LPS for 3 h) and caspase-1 (LPS for 1 h) protein expression were determined using western blotting. GAPDH was used as an internal control for the western blot analyses. Each value indicates the mean ± SD and is representative of results obtained from three independent experiments. # *p* <0.05 vs. untreated group; * *p* < 0.05, ** *p* < 0.01 and *** *p* < 0.001 vs. *P. aeruginosa* LPS group. NT, untreated group; Bay, Bay 11-7082; Par, parthenolide.

We investigated whether BITC attenuates NF-κB nuclear translocation. Western blot images revealed that LPS stimulation of THP-1 cells strongly induces NF-κB p65 nuclear localization and IκBα phosphorylation in the cytosol. However, BITC treatment attenuated LPS-induced NF-κB p65 nuclear localization. Additionally, the phosphorylation of IκBα in the cytosol was significantly inhibited by the BITC treatment of THP-1 cells (Figure 4A). Next, to verify whether the NF-κB p65 signaling pathway is involved in IL-1β production and inflammasome component activation, THP-1 cells were stimulated with *P. aeruginosa* LPS/ATP with or without NF-κB inhibitors (Bay 11-7082 and parthenolide). The upregulated production of IL-1β (Figure 4B) and activation of the inflammasome components NLRP-3 and caspase-1 (Figure 4C) were significantly attenuated by NF-κB inhibitors. These results suggest that attenuation of NF-κB activation by BITC may be the signaling pathway responsible for the inhibition of IL-1β production and inflammasome activation in *P. aeruginosa* LPS/ATP-stimulated THP-1 cells.

## 3. Discussion

The final objectives of this research were to verify whether IL-1β produced by endotoxin-stimulated activation of inflammasomes induces fibrosis and to reveal the reaction mechanism in the growth of nasal polyps (NPs). NPs are a chronic disease of the nose and sinuses with nasal obstruction, runny nose, smell and taste loss, headache, and a high recurrence rate after medical or surgical treatment, often leading to substantial impairment of the quality of life of the patient [19]. IL-1β is expressed in NPs, and *P. aeruginosa* is a common culture isolate in chronic rhinosinusitis (CRS) [20]. In a previous study, NLRP3 was highly expressed in NPs from subjects with CRSwNP [21]. To understand whether fibrosis occurs during NP growth due to IL-1β, first, the production of inflammasome-mediated IL-1β by *P. aeruginosa* LPS/ATP stimulation in macrophages was investigated. Additionally, work was carried out to obtain a substance capable of inhibiting the production of IL-1β via attenuation of inflammasome activation.

NPs are infiltrated by various inflammatory and immune cells, such as plasma cells, lymphocytes, mast cells, eosinophils, neutrophils, and macrophages [22]. Among these cells, macrophages were identified in both the stroma and the epithelium lining the NPs [23]. Macrophages are innate immune effector cells of the innate immune system that play an important role in the recognition and elimination of a foreign pathogen. The primary cytokines produced by macrophages are IL-1β, IL-6, IL-8, IL-33, and TNF-α. THP-1 cells in the monocyte state can be differentiated into macrophage-like cells by induction of PMA. Various macrophage phenotypic intermediates can develop from THP-1 cells during PMA stimulation, showing similarities to primary monocytes and macrophages in terms of morphological and functional properties, including differentiation markers [24]. Therefore, THP-1 cells have been widely used as a suitable in vitro model of human macrophages in mechanistic studies of external stimuli. Macrophages are used as model cell lines to research inflammasome activation for therapeutic and/or mechanistic studies [25]. The upper airways, including the nasopharynx and paranasal sinuses, have been shown to be a silent reservoir for *P. aeruginosa* [26]. Additionally, *P. aeruginosa* was significantly more common in noneosinophilic CRSwNP patients in a sample of Korean adults [27]. Therefore, we used a model that stimulates macrophages with *P. aeruginosa* LPS in this study.

LPS produces IL-1β in an inflammasome-mediated manner in THP-1 cells [28,29]. As expected, the production of IL-1β was significantly increased by *P. aeruginosa* LPS stimulation in our experimental system. However, the increased IL-1β was inhibited in a dose-dependent manner by treatment with 10 and 30 µM BITC without cytotoxicity (Figure 2A). This result suggests that BITC inhibits the IL-1β production by suppressing the *P. aeruginosa* LPS-induced signaling pathways. Several studies have reported that LPS stimulates IL-1β production through the activation of inflammasomes [30,31]. In this regard, we investigated whether BITC inhibits the activation of inflammasomes such as NLRP3 and caspase-1 in the cytosol. As shown in Figure 2B,C, treatment with BITC significantly attenuated the pro-IL-1β and inflammasome (NLRP3 and active caspase-1) activation in a dose-dependent manner and hence may be beneficial in inflammasome-mediated anti-inflammatory response.

Next, we investigated the signaling mechanism of inflammasome activation in differentiated THP-1 macrophages. It is well known that in macrophages, *P. aeruginosa* LPS stimulates MAPKs, including ERK, p38, and JNK and NF-κB pathway activation to upregulate IL-1β production through inflammasome activation [32,33,34]. As shown in Figure 3A, treatment with BITC ameliorated this phosphorylation of ERK and JNK. To verify whether *P. aeruginosa* LPS induces IL-1β production and inflammasome activation through the MAPK signaling pathway, we used specific inhibitors of MAPKs under *P. aeruginosa* LPS stimulation. As expected, our results showed that specific inhibitors of ERK and JNK inhibited IL-1β production (Figure 3B) and inflammasome activation (Figure 3C) in LPS/ATP-stimulated THP-1 cells, suggesting that BITC may be useful for IL-1β production and inflammasome activation through the modulation of ERK and JNK signal activation. Inflammasome activation has been widely implicated in NF-κB signaling [33,35]. Therefore, we examined NF-κB signaling in *P. aeruginosa* LPS-stimulated THP-1 cells. We used western blot analysis to explore NF-κB activation status. When cells are stimulated with LPS, IκBα is phosphorylated and then ubiquitinated, leading to its degradation. In our results, *P. aeruginosa* LPS significantly induced IκBα phosphorylation in the cytosol, but BITC treatment blocked IκBα phosphorylation. In addition, NF-κB was translocated to nuclei by *P. aeruginosa* LPS in THP-1 cells. However, NF-κB nuclear translocation induced by *P. aeruginosa* LPS was attenuated by BITC. To verify whether LPS induces IL-1β production and inflammasome activation through NF-κB activation, we used specific inhibitors of NF-κB (Bay 11-7082 and parthenolide) under *P. aeruginosa* LPS stimulation. Our results showed that specific inhibitors of NF-κB inhibited IL-1β production and inflammasome activation, suggesting that BITC may be useful for IL-1β production and inflammasome activation through the regulation of NF-κB activation. Together with our mechanistic results, the results indicate that BITC attenuated *P. aeruginosa* LPS-stimulated IL-1β production via inflammasome activation by suppressing ERK, JNK, and NF-κB signaling in vitro. Our results are consistent with those of previous publications, showing that BITC exerts a pharmacological effect, which is probably mediated via the inhibition of MAPKs and NF-κB signaling [13,36].

## 4. Materials and Methods

### 4.1. Reagents

We obtained LPS derived from *P. aeruginosa*, dexamethasone (Dex), U 0126, phorbol 12-myristate 13-acetate (PMA), Adenosine triphosphate (ATP), and BITC from Sigma-Aldrich (St. Louis, MO, USA). SP 600126 was obtained from Enzo Life Sciences, Inc. (Farmingdale, NY, USA). BAY 11-7082 and parthenolide were obtained from Santa Cruz Biotechnology Inc. Cell Counting Kit-8 (CCK-8) was obtained from Dojindo Laboratories (Kumamoto, Japan). Antibodies against caspase-1 and pro-IL-1β were obtained from Abcam Inc. (Cambridge, MA, USA). Anti-NF-κB p65 antibodies were purchased from eBioscience (San Diego, CA, USA). Anti-NLRP-3 antibodies were obtained from AdipoGen (San Diego, CA, USA). Antibodies against extracellular signal-related kinase (ERK), phospho (p)-ERK, c-Jun N-terminal kinase (JNK), p-JNK, and p-IκB-α were obtained from Cell Signaling Technology, Inc. (Danvers, MA, USA). Anti-Lamin B antibodies were obtained from Santa Cruz Biotechnology (Santa Cruz, CA, USA). Goat anti-rabbit IgG-horseradish peroxidase was obtained from A-Frontier (Seoul, Korea). The CellTiter-Glo^®^ Luminescent assay was obtained from Promega (Madison, WI, USA).

### 4.2. Cell Culture and Differentiation

The THP-1 human leukemia cell line was purchased from the American Type Culture Collection (Manassas, VA, USA). Cells were cultured in RPMI-1640 medium with 10% FBS. THP-1 cells were differentiated using 50 nM PMA and incubated. After treatment with PMA for 48 h, fresh medium was added for an additional 24 h before *P. aeruginosa* LPS treatment. Cells were checked under a light microscope for evidence of differentiation.

### 4.3. Drug Treatment and Cell Viability Assay

Cells were seeded at 1 × 106 cells/mL and treated with 10 μg/mL *P. aeruginosa* LPS + 5 mM ATP (LPS/ATP) after treatment with the indicated concentrations of BITC or Dex for 1 h in PMA-free conditions. After treatment with *P. aeruginosa* LPS/ATP for 24 h, cellular viability was assessed using the CCK-8 method. We added CCK-8 to each well and incubated it at 37 °C for 1 h, followed by an analysis at 450 nm using a microplate reader (SpectraMax M2e, Molecular Devices, Sunnyvale, CA, USA).

### 4.4. Enzyme-Linked Immunosorbent Assay (ELISA)

After treatment with *P. aeruginosa* LPS/ATP with or without BITC or specific inhibitors in PMA-differentiated THP-1 cells, the level of IL-1β production was measured using an ELISA kit (R&D Systems, Minneapolis, MN, USA). The ELISA results were quantified using an ELISA plate reader (SpectraMax M2e) at 450 nm with a correction of 540 nm.

### 4.5. Western Blot Analysis

PMA-differentiated THP-1 cells were stimulated with *P. aeruginosa* LPS with or without pretreatment with BITC or specific inhibitors. Proteins were collected using a NE-PER nuclear and cytoplasmic extraction reagent kit (Pierce, Rockford, IL, USA). Equal quantities of protein were separated on 10% SDS-polyacrylamide minigels and transferred to nitrocellulose membranes. Incubation with the appropriate primary antibodies was performed overnight at 4 °C. Then, the membranes were incubated for 1 h at room temperature with a secondary antibody (goat anti-rabbit IgG and goat anti-mouse IgG) conjugated to horseradish peroxidase. Following three washes in Tris-buffered saline Tween-20 (TBST 20), the immunoreactive bands were visualized using an ECL detection system (Pierce, Rockford, IL, USA). Quantitative data were obtained by densitometry analysis using Multi Gauge version 2.2 software (Fuji Film, Tokyo, Japan).

### 4.6. Statistical Analysis

Data are presented as the mean ± SD. All statistical analyses were performed using GraphPad Prism software 5.0 (GraphPad Software Inc., La Jolla, CA, USA). Comparisons between groups were performed by Dunnett’s multiple range tests. Values of *p* < 0.05 were considered to be statistically significant.

## 5. Conclusions

In this study, our data demonstrated that BITC treatment suppresses IL-1β production via inhibition of inflammasome activation in *P. aeruginosa* LPS-stimulated THP-1 macrophages. In addition, these inhibitory activities of BITC were mediated through the modulation of both the MAPK and NF-κB signaling pathways. Thus, BITC may be a useful novel therapy for LPS-associated inflammatory pathological conditions, including nasal polyps (NP), by targeting inflammasome-mediated IL-1β production signaling pathways. In the next study, we will conduct further investigation to determine whether BITC suppresses IL-1β -induced fibrosis in nasal-polyp-derived fibroblasts (NPDFs) via the excessive accumulation of cellular sources of extracellular matrix (ECM) proteins, which are hallmarks of fibrosis, such as collagen-1 and fibronectin, that could be involved in the growth of NPs. Then, using BITC, we plan to conduct ex vivo experiments on the effect of NP growth. Therefore, fibrosis is triggered by inflammasome-mediated IL-1β expression activated after *P. aeruginosa* LPS stimulation, and we plan to verify the efficacy of BITC for regulating NP growth.

## Data Availability

Not applicable.
